# Gut microbiome and metabolic health: mechanisms and precision interventions

**DOI:** 10.1080/19490976.2026.2644677

**Published:** 2026-04-21

**Authors:** Zhengrui Li, Sudeshna Samui, Ji'an Liu, Yang Yang, Xue Liu, Qingyu Chen, Jing Li, Divya Gopinath, Peng Luo, Dan Shan

**Affiliations:** a Shanghai Jiao Tong University School of Medicine, Shanghai, People's Republic of China; b Hooghly Women's College, University of Burdwan, West Bengal, India; c Heilongjiang University of Traditional Chinese Medicine, Harbin, People's Republic of China; d The Second School of Medicine, Wenzhou Medical University, Wenzhou, People's Republic of China; e Shanghai Stomatological Hospital & School of Stomatology, Fudan University, Shanghai, People's Republic of China; f Basic Medical and Dental Sciences Department, College of Dentistry, Ajman University, Ajman, UAE; g Centre of Medical and Bio-allied Health Sciences Research, Ajman University, Ajman, UAE; h Department of Oncology, Zhujiang Hospital, Southern Medical University, Guangzhou, People's Republic of China; i Health Innovation One, Sir John Fisher Drive, Lancaster University, Lancaster, UK

**Keywords:** Gut microbiome, metabolic health, short-chain fatty acids, gut barrier function, obesity and type 2 diabetes, microbiome-targeted interventions, precision microbiome medicine

## Abstract

The gut microbiome is increasingly recognized as a fundamental regulator of metabolic health, shaping energy balance, insulin sensitivity, inflammatory tone, and inter-organ communication through a broad spectrum of microbial metabolites that engage host signaling pathways. In this review, we synthesize current mechanistic insights into how gut microbial communities shape metabolic function, with particular emphasis on short-chain fatty acids, secondary bile acid signaling, gut barrier integrity, immune modulation, and the microbiota–gut–brain–pancreas axis. We further summarize disease-associated alterations in microbial composition and function across obesity, type 2 diabetes, metabolic dysfunction-associated steatotic liver disease, and metabolic syndrome, highlighting key microbial and metabolic features that contribute to metabolic dysfunction. Evidence from germ-free models, fecal microbiota transplantation studies, and strain-level interventions suggests that shifts in microbial ecology may causally shape metabolic outcomes. We also critically evaluate emerging microbiome-centered therapeutic strategies, including targeted probiotics, prebiotics, dietary modulation, and fecal microbiota transplantation, while addressing factors that underlie inter-individual variability in treatment responses. In addition, we discuss the growing influence of multi-omics technologies, microbial metabolic modeling, and machine learning approaches in advancing precision microbiome medicine. To integrate these advances within a coherent framework, we outline a precision microbiome intervention pipeline linking multidimensional profiling to functional stratification and targeted therapeutic design. We also introduce a conceptual Precision Microbiome Intervention Triangle to mechanistically explain heterogeneity in responses to microbiome-targeted therapies. Collectively, these insights establish and position the gut microbiome as both a mechanistic driver and a modifiable therapeutic target in metabolic disease, and highlight key challenges and future directions for the development of personalized microbiome-based metabolic interventions.

## 1. Introduction

Metabolic diseases, including obesity, type 2 diabetes (T2D), metabolic dysfunction-associated steatotic liver disease (MASLD), and metabolic syndrome (MetS), have escalated globally and now constitute a major threat to public health.^1-3^ Conventional therapeutic strategies, ranging from lifestyle modification to pharmacologic intervention, have shown limited long-term efficacy, underscoring the need for new mechanistic frameworks and precision-targeted approaches.^4-7^ In this context, the gut microbiome has emerged as a central regulator of metabolic homeostasis,^6^^,^^8^^,^^9^ influencing energy balance, glucose control, lipid metabolism, inflammatory tone, and organ-to-organ communication via a diverse inventory of microbial metabolites and host signaling pathways.^7^^,^^8^^,^^10-18^ Recent assessments of post-COVID and long COVID–related disability highlight substantial and persistent health burdens,^19^^,^^20^ underscoring how underlying physiological vulnerabilities may influence long-term outcomes and reinforcing the importance of mechanistic research into host–microbiome interactions.

Over the past decade, advances in metagenomics, metabolomics, and microbial ecology have unveiled a complex “microbiome–metabolism axis” through which gut microbes shape host physiology.^6^^,^^7^^,^^17^^,^^21^^,^^22^ These discoveries have highlighted multiple mechanistic routes, including short-chain fatty acid (SCFAs) production, secondary bile acid signaling, maintenance of intestinal barrier integrity, and interactions along the “microbiota–gut–brain–pancreas axis”.^14^^,^^23-25^ Dysbiosis, marked by reduced microbial diversity, loss of beneficial taxa, and expansion of pro-inflammatory species, has been consistently linked to metabolic disorders across human cohorts and preclinical models.^7^^,^^8^^,^^26^ Importantly, fecal microbiota transplantation (FMT) and germ-free animal studies provide evidence that alterations in microbial communities can causally influence adiposity, insulin sensitivity, and hepatic lipid homeostasis, emphasizing the functional significance of the microbiome in metabolic disease pathogenesis.^14^^,^^27^^,^^28^

Yet despite these advances, several critical gaps remain. The precise molecular targets of microbial metabolites, the strain-level ecological interactions that govern metabolic outputs, and the determinants of interindividual heterogeneity in microbiome–metabolism relationships are only beginning to be elucidated.^23^^,^^29^^,^^30^ Additionally, while microbiome-directed interventions such as probiotics, prebiotics, dietary modulation, and FMT show promise, therapeutic responses vary widely among individuals—highlighting the need for mechanistic clarity and precision microbiome medicine.^22^^,^^31^^,^^32^

Together, these considerations establish a definitive need for an integrated synthesis of how the gut microbiome regulates metabolic health, how dysbiosis contributes to specific metabolic diseases, and how emerging interventions may be optimized for clinical translation.^14^^,^^17^^,^^22^ In this review, we consolidate current mechanistic insights into microbiome–host metabolic interactions, evaluate evidence linking the gut microbiome to major metabolic disorders, and assess emerging strategies for microbiome-based therapy. We further outline key challenges, including causal inference, strain-level specificity, interindividual variability, and safety considerations, and discuss future directions toward personalized, microbiome-centered metabolic interventions.

**Figure 1.**
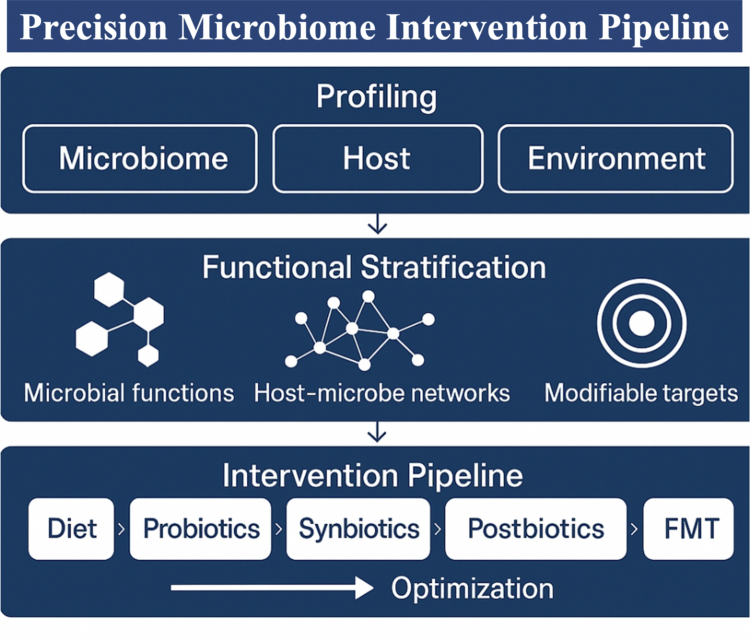
A precision microbiome intervention pipeline integrating profiling, functional stratification, and targeted therapeutic design.

To provide a coherent structure for this synthesis, the conceptual organization of this review follows a translational pipeline aligned with the emerging framework of precision microbiome medicine (Figure 1). We first outline multi-layer profiling strategies encompassing microbial, host, and environmental determinants that collectively define metabolic phenotypes. We then discuss functional stratification approaches that integrate microbial metabolic capacities, host–microbe interaction networks, and modifiable ecological or molecular targets. Building on these mechanistic foundations, we evaluate current and emerging intervention modalities, including diet, probiotics, synbiotics, postbiotics, and FMT, with emphasis on how these strategies can be optimized and personalized. This framework provides a translational roadmap that connects mechanistic insight to clinical application and underpins the structure of the sections that follow. Rather than presenting a catalog of existing tools, Figure 1 organizes widely used approaches into a decision-oriented, iterative pipeline featuring sequential decision points and feedback loops that enable continuous refinement of microbiome-targeted interventions toward precision implementation. In addition, we propose a Precision Microbiome Intervention Triangle to mechanistically explain inter-individual variability in responses to microbiome-targeted therapies.

The precision microbiome intervention pipeline provides a conceptual roadmap linking multi-dimensional profiling to mechanistically informed therapeutic strategies. The framework begins with comprehensive profiling of the gut microbiome, host factors, and environmental influences that shape metabolic phenotypes. To enhance feasibility and standardization, environmental influences can be conceptualized through a tiered framework, ranging from routinely collected data on diet, lifestyle, and medication use, to wearable and metabolomic indicators of exposure, and, where appropriate, broader exposomic measures such as air pollution or occupational risk. Functional stratification integrates microbial metabolic pathways, host–microbe interaction networks, and modifiable ecological or molecular targets to define actionable biological mechanisms. These insights inform a spectrum of intervention strategies, including dietary modulation, probiotics, synbiotics, postbiotics, and FMT, that can be iteratively optimized to enhance efficacy and personalize treatment. Together, this pipeline illustrates how ecological and mechanistic understanding of the microbiome can be translated into precision interventions for metabolic disease.

## 2. The fundamental link between the gut microbiome and metabolic health

### 2.1. Characteristics of a metabolically healthy gut microbiome

A metabolically healthy gut microbiome is defined not only by its taxonomic composition but also by its functional capacity and ecological resilience.^14^^,^^15^^,^^21^ From a taxonomic standpoint, high *α*-diversity is widely considered a hallmark of microbial stability, enabling the community to maintain metabolic functions despite dietary or environmental fluctuations.^7^^,^^21^ Beneficial taxa, including *Akkermansia muciniphila (A. muciniphila), Faecalibacterium prausnitzii (F. prausnitzii), Bifidobacterium*, and other SCFA-producing organisms, play a central role in supporting intestinal barrier integrity, modulating immune responses, and maintaining balanced energy metabolism.^8^^,^^10^^,^^11^

From a functional perspective, the core functional genome shared across healthy individuals includes conserved pathways involved in carbohydrate fermentation, SCFA biosynthesis, and bile acid transformation.^7^^,^^14^^,^^16^ SCFAs such as butyrate, acetate, and propionate provide energy substrates for colonocytes, regulate lipid and glucose metabolism, and exert broad anti-inflammatory effects.^33-35^ A balanced Firmicutes-to-Bacteroidetes ratio further contributes to efficient energy harvesting and metabolic flexibility, although the specific directionality of this ratio varies across populations and contexts.^7^^,^^36^ Importantly, mucin-degrading bacteria such as *A. muciniphila* help maintain the mucosal layer and promote gut barrier function, reducing systemic inflammation and supporting metabolic homeostasis.^11^^,^^14^

**Figure 2.**
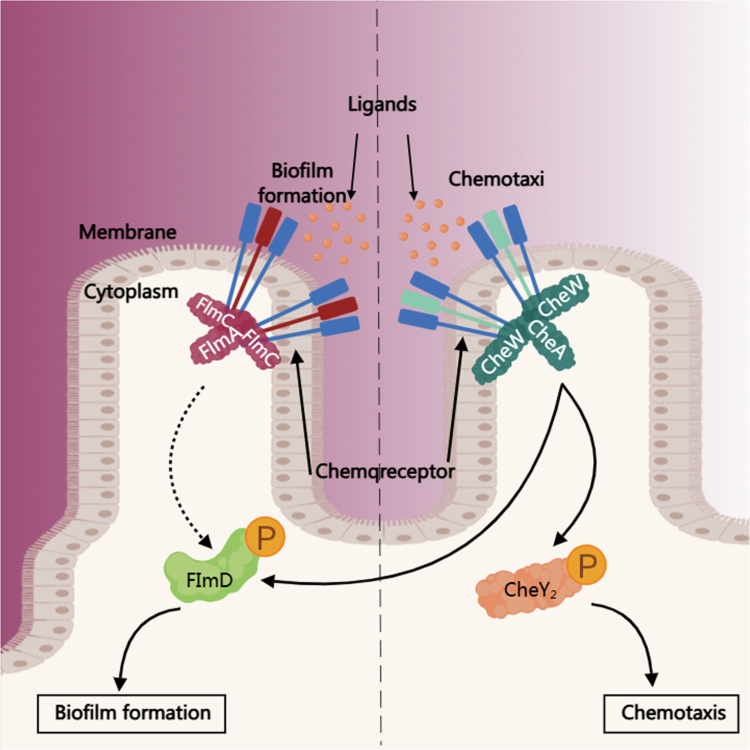
Chemotaxis and biofilm-mediated ecological processes underlying gut bacterial colonization.

Beyond taxonomic and functional features, the ability of gut bacteria to establish stable ecological niches is essential for maintaining a resilient and metabolically competent microbial community. Processes such as chemotaxis-driven niche localization and biofilm-mediated adhesion enable commensal species to sense environmental cues, anchor to intestinal surfaces, and persist over time, thereby forming the ecological foundation for downstream microbial metabolic activity and host–microbe interactions (Figure 2).^22^^,^^37^

Commensal gut bacteria employ coordinated chemotaxis systems (such as the Che signaling pathway) and biofilm-associated machinery (including the Fim operon) to sense environmental ligands, adhere to intestinal surfaces, and migrate toward favorable ecological niches. These colonization behaviors enable the establishment and long-term maintenance of stable microbial communities. Such ecological stability provides the foundational conditions that support host–microbe interactions and allow the gut microbiome to sustain consistent metabolic outputs that contribute to overall metabolic health.

### 2.2. Alterations in microbial composition and function in metabolic disorders

Metabolic disorders, including obesity, T2D, MASLD, and MetS, are consistently associated with dysbiosis characterized by reduced microbial diversity, loss of beneficial taxa, and enrichment of pro-inflammatory or metabolically deleterious species.^7^^,^^8^^,^^26^ These compositional shifts are accompanied by shared functional impairments, including diminished production of SCFAs, disrupted bile acid transformation, compromised gut barrier integrity, and heightened inflammatory signaling, all of which directly contribute to metabolic dysregulation.^7^^,^^16^

Across diverse metabolic conditions, dysbiosis is commonly linked to impaired energy homeostasis, chronic low-grade inflammation, and insulin resistance, reflecting convergent downstream consequences despite heterogeneity in upstream microbial signatures.^7^^,^^33^^,^^38^ Reduced *α*-diversity and expansion of lipopolysaccharide (LPS)-producing taxa promote metabolic endotoxemia, which acts as a unifying mechanistic driver of systemic inflammation, hepatic lipid accumulation, and impaired glucose metabolism across disease states.^32^^,^^39^^,^^40^

Collectively, these alterations indicate that loss of beneficial microbial functions and expansion of pro-inflammatory ecological networks represent core features underlying metabolic impairment. While individual metabolic diseases exhibit distinct taxonomic patterns and disease-specific microbial associations, these shared dysbiotic features form a common mechanistic framework.^7^^,^^8^^,^^16^

### 2.3. Evidence supporting a causal role of the gut microbiome in metabolic regulation

Compelling evidence from germ-free models, FMT studies, and strain-level interventions indicates that the gut microbiome plays a causal role in shaping metabolic outcomes. Germ-free mice display altered energy balance, impaired glucose tolerance, and abnormal lipid metabolism, all of which can be reversed or modulated by introducing specific microbial communities.^14^^,^^15^^,^^17^ Colonization with microbiota from healthy donors improves metabolic parameters, whereas transplantation of microbiota from obese or insulin-resistant individuals induces increased adiposity, hepatic steatosis, and impaired insulin sensitivity in germ-free recipients.^9^^,^^10^^,^^27^

FMT studies in humans further support a causal link, showing that transfer of microbiota from lean donors can transiently improve insulin sensitivity in individuals with MetS.^28^^,^^41^ Strain-level interventions highlight the mechanistic specificity of microbial effects: for example, supplementation with *A. muciniphila* enhances gut barrier function, reduces inflammation, and improves glucose homeostasis in both animal models and early-stage clinical trials. Similar effects have been observed with targeted *Bifidobacterium* and *Lactobacillus* strains, which modulate bile acid pools, SCFA production, and immune signaling pathways.^29^^,^^42^^,^^43^

Collectively, these experiments demonstrate that microbial communities and even individual strains can modulate host metabolism in a directionally consistent and mechanistically interpretable manner, reinforcing the microbiome's role as a causal rather than merely associative factor in metabolic disease pathogenesis.^14^^,^^17^^,^^22^

## 3. Key mechanisms linking the gut microbiome to metabolic regulation

The gut microbiome influences host metabolism through several interconnected mechanisms that involve microbial metabolites, regulation of intestinal barrier integrity, immune and inflammatory signaling, and neuroendocrine communication along the microbiota–gut–brain–pancreas axis.^14^^,^^15^^,^^23^ Together, these pathways shape energy balance, glucose homeostasis, lipid metabolism, and systemic inflammation, thereby providing mechanistic links between microbial community structure and metabolic health.^7^^,^^16^

### 3.1. Microbial metabolites as central mediators of host metabolism

Microbial metabolites are key intermediates that translate microbial ecological activity into host metabolic effects. Among these, SCFAs, secondary bile acids, and endotoxin-related molecules such as LPS have been most intensively studied.^12^^,^^33^^,^^39^

SCFAs, including acetate, propionate, and butyrate, are produced through the fermentation of dietary fibers and resistant starches.^33^^,^^34^ These metabolites act as major energy substrates for colonocytes, support mitochondrial function, and contribute to the maintenance of gut barrier integrity. SCFAs also signal through G protein–coupled receptors such as GPR41 and GPR43 to regulate appetite, energy expenditure, and insulin sensitivity, and they can inhibit histone deacetylases to modulate gene expression in metabolic tissues.^33^^,^^44^^,^^45^ Prior experimental studies using receptor-knockout models showed that loss of GPR41 or GPR43 signaling may blunt the metabolic benefits of SCFAs, providing more evidence that these receptors could mediate microbe–host metabolic communication.^44^^,^^46^ Reduced SCFA production, often observed in dysbiosis associated with obesity and T2D, is linked to impaired glucose homeostasis and increased inflammation.^35^

Gut bacteria also transform primary bile acids into secondary bile acids that activate host receptors including Farnesoid X receptor (FXR) and TGR5.^12^^,^^38^^,^^47^ Animal models in which microbial enzymes involved in bile-acid transformation are selectively inhibited suggested impaired FXR and TGR5 activation, potentially supporting a causal role for microbially mediated bile-acid remodeling in metabolic regulation.^48^^,^^49^ FXR–bile acid signaling regulates hepatic gluconeogenesis, lipid metabolism, and cholesterol homeostasis, whereas TGR5 activation promotes energy expenditure, incretin secretion, and anti-inflammatory effects. Dysbiosis can disrupt bile acid pools and receptor activation, contributing to IR, hepatic steatosis, and dyslipidemia.^32^^,^^38^^,^^47^ Restoring microbial taxa that support beneficial bile acid transformations has been shown to improve metabolic parameters in preclinical models.^50^^,^^51^

In contrast to these beneficial metabolites, LPS and related endotoxin molecules derived from gram-negative bacteria promote chronic low-grade inflammation when they reach the systemic circulation.^23^^,^^39^ LPS activates pattern recognition receptors such as TLR4, driving pro-inflammatory cytokine production in adipose tissue, liver, and vasculature. This state of “metabolic endotoxemia” is associated with IR, hepatic inflammation, and cardiovascular risk, and is exacerbated by increased gut permeability and overgrowth of LPS-producing taxa.^23^^,^^39^^,^^40^ A randomized-controlled clinical trial reducing gut-derived endotoxin load suggested parallel improvements in insulin sensitivity and inflammatory tone, suggesting that metabolic endotoxemia may not be merely correlative but functionally contributes to metabolic impairment.^52^

Beyond macronutrient-derived metabolites, the microbiome also modulates micronutrient and xenobiotic metabolism in ways that influence metabolic health. Certain bacterial species sequester dietary iron through siderophore production or specialized uptake systems, altering its bioavailability to the host. Both iron deficiency and overload have been linked to impaired glucose regulation and increased cardiovascular risk, suggesting that microbially mediated iron handling represents an additional route through which the microbiome affects metabolic outcomes.^53-55^ Beyond iron, disturbances in other trace metal pathways may also influence host metabolic signaling. Dysregulated copper homeostasis, for example, was shown to reshape redox balance, immune activation, and metabolic networks in cancer, underscoring the broader relevance of micronutrient-regulated metabolic pathways.^56^ Likewise, the microbiome participates in the biotransformation of non-nutritive sweeteners (NNS). Although NNS contain little or no energy, several studies indicate that they may reshape gut microbial communities and, in some contexts, promote glucose intolerance and metabolic dysfunction.^57-59^ These examples underscore that the metabolic impact of dietary components depends not only on their caloric content but also on their interactions with gut microbes. Beyond classical metabolic pathways, microbiome-derived metabolites also influence distant physiological systems. For example, recent research highlights a gut–bone axis in which microbial metabolites and immune signaling modulate bone turnover and musculoskeletal health,^60^ further illustrating the systemic reach of microbiome-driven metabolic regulation.

Collectively, these metabolite classes converge on three major mechanistic layers, receptor-mediated signaling (GPR41/43, FXR, and TGR5), inflammatory activation via TLR4-driven endotoxemia, and epigenetic regulation through HDAC inhibition, that jointly shape host glucose and lipid homeostasis.^33^

### 3.2. Microbial regulation of intestinal barrier function and systemic inflammation

The intestinal barrier is a critical interface that separates the luminal microbial ecosystem from the internal milieu. The gut microbiome plays a central role in maintaining barrier integrity, thereby limiting the translocation of microbial products and preventing excessive systemic inflammation.^40^^,^^61^

One key mechanism involves the regulation of the mucus layer, which is composed primarily of mucins secreted by goblet cells. Commensal and probiotic bacteria can stimulate mucin production and support a thick, continuous mucus layer that physically separates luminal microbes from epithelial cells. Species such as *A. muciniphila* contribute to controlled mucin turnover and help maintain a balanced mucosal environment. Loss of these beneficial taxa, or overgrowth of aggressive mucus-degrading organisms, can thin the mucus layer and predispose to barrier disruption.^11^^,^^14^ In addition, oral-derived pathobionts such as *Fusobacterium* have been shown to translocate along the oral–gut axis and disrupt mucosal integrity, promoting epithelial inflammation and barrier breakdown. These findings highlight how the expansion of specific pro-inflammatory taxa may exacerbate permeability-related metabolic disturbances.^62^

The microbiome also modulates the expression and organization of tight junction proteins, including occludin, claudins, and ZO-1, which govern paracellular permeability.^24^^,^^63^ Dysbiosis is frequently associated with reduced tight junction expression and increased intestinal permeability, often referred to as “leaky gut.” A compromised barrier allows luminal endotoxins such as LPS and other microbial metabolites to enter the systemic circulation, triggering inflammatory cascades that contribute to IR, endothelial dysfunction, and hepatic injury.^32^^,^^40^

The relationship between barrier dysfunction and systemic inflammation is further highlighted by the gut–liver axis.^64^ Microbial products that reach the liver via the portal vein can overwhelm hepatic detoxification pathways and promote hepatic inflammation, steatosis, and progression toward MASLD.^65^ In parallel, systemic dissemination of microbial antigens activates pattern recognition receptors in adipose tissue and vasculature, amplifying chronic low-grade inflammation that underlies many metabolic disorders.^66-68^

Restoring barrier integrity through microbiome-targeted interventions has shown promise in both preclinical and clinical studies. Specific probiotic strains enhance tight junction expression and mucosal integrity, while dietary fibers and resistant starches promote the expansion of SCFA-producing bacteria that reinforce epithelial health and reduce permeability-associated inflammation.^43^^,^^69^^,^^70^ These findings position the microbiome–barrier–inflammation axis as a key mechanistic link between dysbiosis and metabolic disease, and as an attractive target for therapeutic intervention.

### 3.3. Microbial regulation of the gut–brain axis and the gut–brain–pancreas axis

**Figure 3.**
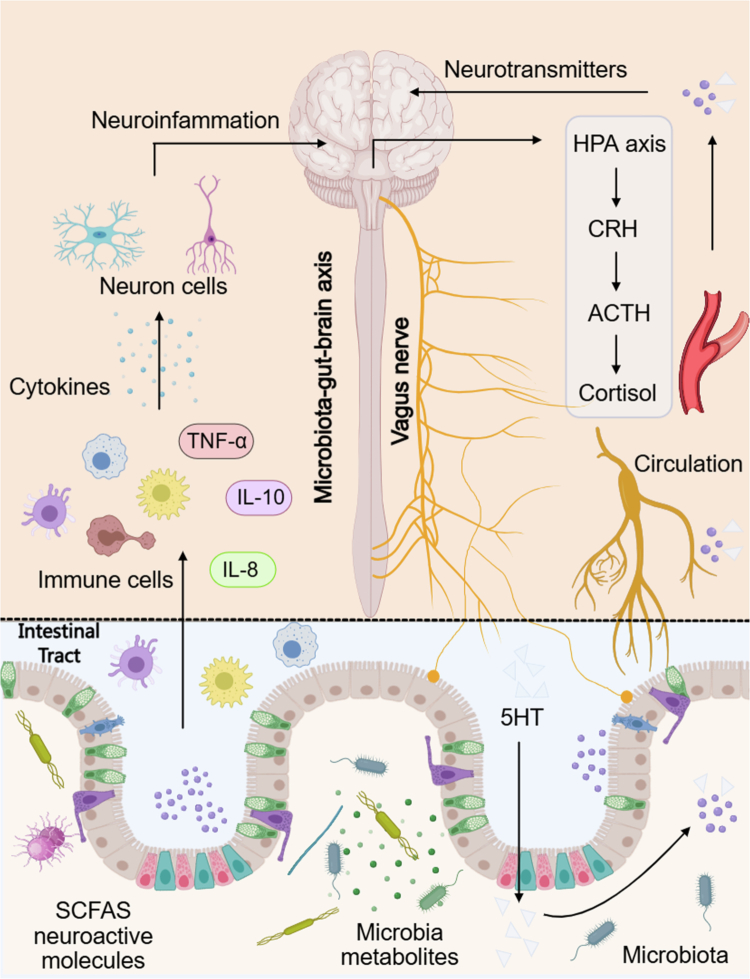
Microbiota–gut–brain communication pathways integrating neural, immune, and endocrine signaling.

The microbiota–gut–brain axis represents a complex bidirectional communication network through which gut microorganisms influence central nervous system function and systemic metabolic regulation.^25^^,^^71^^,^^72^ Through the production of microbial metabolites, neuroactive compounds, and immune-modulatory signals, gut microbes actively shape neural activity, appetite regulation, stress responsiveness, and neuroinflammatory processes. These signals are transmitted to the brain via both neural pathways, particularly vagal afferent fibers, and humoral routes involving circulating metabolites and cytokines, thereby engaging brainstem and hypothalamic circuits that govern energy balance and neuroendocrine homeostasis (Figure 3).

Gut microbes produce a variety of neuroactive compounds, including *γ*-aminobutyric acid (GABA), serotonin, and other neurotransmitter precursors, as well as SCFAs that can activate vagal afferent pathways.^25^^,^^39^^,^^73^ In parallel, microbiota-driven modulation of intestinal immune cells influences cytokine production, which can affect neuronal activity and contribute to neuroinflammatory signaling. Together, these neural and immune pathways enable the gut microbiota to influence central processes related to appetite, mood, stress responses, and broader neurological function.

Within this broader microbiota–gut–brain axis, a distinct and metabolically relevant gut–brain–pancreas communication loop can be delineated. Microbial signals processed at the level of the central nervous system can modulate autonomic output to the pancreas, thereby influencing pancreatic *β*-cell function, insulin secretion, and glucose homeostasis.^25^^,^^39^ Conversely, alterations in pancreatic endocrine activity may feedback to the gut environment through changes in nutrient availability and gastrointestinal motility. Dysbiosis-associated disruption of this gut–brain–pancreas triangle has been linked to impaired insulin secretion, reduced glucose tolerance, and decreased insulin sensitivity, collectively contributing to an increased risk of obesity and T2D.^39^^,^^74^^,^^75^

Experimental studies further support the functional importance of this neuro–microbial–endocrine circuitry, as vagal stimulation has been shown to improve insulin sensitivity and promote *β*-cell proliferation in preclinical models.^76-79^ SCFAs, particularly butyrate and propionate, can directly or indirectly activate vagal afferents and influence central energy-regulatory centers, while also modulating pancreatic *β*-cell responsiveness.^80-84^

Collectively, these neuroendocrine pathways provide a mechanistic link between microbial signaling and clinically measurable metabolic outcomes, including insulin dynamics, glucose tolerance, and systemic energy homeostasis, thereby highlighting the translational relevance of the ‘Gut–Brain–Pancreas’ axis in metabolic disease.

Understanding how microbial metabolites and taxa shape the gut–brain–pancreas axis provides important insight into the neuroendocrine control of metabolism and suggests novel therapeutic strategies.^14^^,^^85^^,^^86^ Microbiota-directed interventions, dietary modulation, and neuromodulation strategies targeting this axis represent promising avenues to restore metabolic control in individuals with dysbiosis-associated metabolic disease (Figure 3).

Gut microbiota–derived metabolites, including SCFAs and neuroactive molecules, interact with the intestinal epithelium and enteroendocrine signaling pathways, leading to the release of neurotransmitters such as serotonin (5-HT). Microbial signals are conveyed to the central nervous system through vagal afferent fibers and via the systemic circulation. In parallel, microbiota-driven modulation of intestinal immune cells influences cytokine production, including TNF-*α*, IL-8, and IL-10, which can affect neuronal activity and neuroinflammatory processes. Within the brain, these integrated neural and immune inputs engage central regulatory networks and the hypothalamic–pituitary–adrenal (HPA) axis, resulting in downstream endocrine responses characterized by CRH, ACTH, and cortisol release. Collectively, this microbiota–gut–brain axis illustrates how gut microbial metabolism shapes neuroendocrine signaling and systemic physiological homeostasis.

## 4. The association between the gut microbiome and specific metabolic diseases

The gut microbiome has emerged as a central regulator of metabolic health, with extensive evidence linking its composition and function to the development of major metabolic diseases.^7^^,^^14^ MetS is characterized by central obesity, IR, dyslipidemia, and hypertension, and is strongly associated with gut microbial dysbiosis.^8^^,^^26^ Alterations in microbial diversity and taxonomic composition can promote systemic inflammation, impair insulin signaling, and disrupt lipid metabolism, which are core features of MetS and related disorders.^11^^,^^33^

Specific microbial taxa correlate with metabolic status.^21^^,^^36^ Shifts in the relative abundance of Firmicutes and Bacteroidetes associate with adiposity and insulin sensitivity, while depletion of beneficial species such as *A. muciniphila* and *F. prausnitzii* is linked to greater metabolic impairment. These patterns support the use of microbiome profiling as a potential diagnostic or prognostic tool for assessing metabolic risk.^28^^,^^87^

Microbial metabolites, particularly SCFAs, play a key mechanistic role. SCFAs enhance insulin sensitivity, regulate appetite and energy expenditure, and support gut barrier integrity.^33^^,^^35^ Western-style diets, typically low in fiber and high in ultra-processed foods, reduce SCFA production and favor dysbiosis, thereby exacerbating metabolic dysfunction.^6^^,^^70^ Conversely, fiber-rich dietary patterns promote SCFA producers and are associated with improved metabolic outcomes.

Beyond MetS, T2D, MASLD, and hypertension have each been linked to distinct patterns of gut dysbiosis and altered microbial function.^75^^,^^88^ Metabolic modeling suggests that T2D microbiomes display increased autonomy and reduced host-to-microbiome metabolic flux, reflecting a decoupling of host and microbial metabolism. At the same time, the microbiome–metabolism relationship is bidirectional. Host physiological states can reshape microbial communities, as illustrated by individuals with chronic spinal cord injury whose gut dysbiosis resembles that seen in MetS, raising questions about cause and consequence in dysbiosis.

Ethnic and population differences in metabolic disease prevalence and progression may also be partly mediated by microbiome variation.^89^^,^^90^ Distinct dietary patterns, host genetics, and microbial configurations contribute to heterogeneous metabolic phenotypes across populations, underscoring the need for microbiome-informed precision strategies in metabolic disease prevention and treatment.

### 4.1. Obesity

The relationship between the gut microbiome and obesity involves multiple interconnected pathways that influence energy balance, adipose tissue function, and systemic inflammation.^10^^,^^11^ One well-described mechanism is enhanced energy extraction from the diet. Individuals with obesity often display an altered Firmicutes-to-Bacteroidetes ratio and enrichment of taxa that are efficient at harvesting energy from otherwise non-digestible carbohydrates, contributing to greater caloric yield and fat storage.^9^^,^^91^

Dysbiosis also promotes chronic low-grade inflammation and IR, key drivers of obesity-related metabolic dysfunction.^11^^,^^33^ Gut-derived pro-inflammatory signals and endotoxins can act on adipose tissue to amplify cytokine production and macrophage infiltration, worsening systemic IR.^40^^,^^63^ Changes in microbial metabolites, including reduced SCFAs and altered bile acid profiles, further modulate inflammatory tone and lipid handling.^33^^,^^92^

The microbiome influences endocrine regulators of appetite and energy expenditure. Alterations in microbial composition have been associated with leptin resistance and disrupted satiety signaling, promoting hyperphagia.^93^ SCFAs and other microbial metabolites can modulate gut hormone secretion and hypothalamic circuits that govern feeding behavior.^33^^,^^93^ Interventions that enrich SCFA-producing bacteria, including targeted probiotics and prebiotic fibers, have been shown to improve leptin sensitivity, reduce body weight, and ameliorate metabolic parameters in experimental and clinical studies.^43^^,^^94^

Importantly, the gut microbiome may help explain why individuals respond differently to the same dietary interventions.^6^^,^^95^ Microbiome composition influences the metabolic and inflammatory consequences of specific macronutrient patterns, leading to variable weight loss and cardiometabolic responses across individuals exposed to identical diets.^6^^,^^95^ This variability supports the development of precision nutrition strategies in which diet is tailored to an individual’s microbial profile to maximize efficacy and limit obesity-related complications.^96^^,^^97^

Overall, the microbiome–obesity axis integrates altered energy harvest, immune activation, and hormonal regulation. Continued work to link specific taxa and functional pathways to obesity phenotypes will facilitate the design of microbiome-targeted approaches that complement existing lifestyle and pharmacologic therapies.

### 4.2. Type 2 diabetes

T2D is characterized by IR, impaired beta-cell function, and chronic low-grade inflammation, all of which are influenced by the gut microbiome.^74^^,^^88^ T2D-associated dysbiosis often includes reduced microbial diversity, depletion of beneficial SCFA producers, and enrichment of taxa that promote inflammation and metabolic stress.

One key pathway involves SCFA-mediated regulation of insulin sensitivity. SCFAs produced during fiber fermentation can stimulate incretin secretion, enhance insulin release from pancreatic beta-cells, and improve peripheral insulin action.^33^^,^^35^ Recent human multi-omics analyzes suggest that variation in circulating SCFA levels may partially reflect microbial metabolic capacity and could relate to differences in insulin sensitivity across individuals.^98-100^ Dysbiosis that reduces SCFA output or alters SCFA profiles contributes to impaired glucose regulation. Certain taxa, such as *Akkermansia* and *Faecalibacterium*, associate with more favorable metabolic profiles, while others, including *Ruminococcus gnavus (R. gnavus)*, correlate with more severe IR.^74^^,^^101^

Microbiome-targeted therapies may protect beta-cell function as well as improve insulin sensitivity. SCFAs such as butyrate can suppress inflammatory signaling and oxidative stress in beta-cells, preserving their secretory capacity.^33^ FMT from metabolically healthy donors has shown promise in early studies, with improvements in insulin sensitivity and glycemic control in some individuals with T2D.^27^^,^^41^

Emerging work highlights the value of microbial and metabolic biomarkers for early detection. Distinct gut microbiome signatures have been identified in individuals with prediabetes, including enrichment of operational taxonomic units from genera such as *Prevotella and Megasphaera*, which may predict progression toward overt T2D.^89^ Enterotype patterns, including *Bacteroides*-dominant communities, have also been associated with increased diabetes risk in some cohorts.^102^ Multi-omics approaches integrating microbiome, metabolome, and host data are beginning to define biomarker panels for precision diagnosis and risk stratification in prediabetes.^103^^,^^104^

Metabolic modeling further demonstrates that T2D microbiomes exhibit increased metabolic independence and antagonism.^105^ Reduced host-to-microbiome metabolic flux suggests weakened mutualistic exchange, which may exacerbate IR and metabolic inflexibility. Together, these findings position the gut microbiome as both a modifiable driver and a potential biomarker source in the continuum from prediabetes to overt T2D.

### 4.3. Metabolic dysfunction-associated steatotic liver disease

The gut–liver axis is central to the pathogenesis of MASLD, a growing global health burden tightly linked to obesity and MetS.^26^^,^^32^ Bidirectional communication between the intestinal microbiome and the liver occurs through microbial metabolites, bile acids, and translocated microbial products that reach the liver via the portal circulation.^47^^,^^92^

The gut microbiota regulates hepatic lipid metabolism through SCFAs and bile acids that influence lipid storage, oxidation, and inflammatory pathways.^38^^,^^47^ Specific taxa, such as *R. gnavus*, have been positively associated with hepatic fat accumulation, suggesting that compositional shifts in the microbiome can drive steatosis.^101^ Dysbiosis is commonly accompanied by increased intestinal permeability, which permits endotoxins such as LPS to enter the portal vein and trigger hepatic inflammation, promoting progression from simple steatosis to metabolic dysfunction-associated steatohepatitis (MASH).^40^^,^^63^

Bile acid metabolism is another critical interface. Bile acids synthesized in the liver and modified by gut bacteria act as signaling molecules that activate receptors including FXR, which regulate bile acid homeostasis, lipid metabolism, and glucose balance.^47^^,^^92^ Studies integrating bile acid profiling with microbiome sequencing in metabolic disease cohorts have identified associations between specific secondary bile acids and IR, suggesting potential pathways that merit further mechanistic validation.^106^^,^^107^ However, the degree to which BA–microbiota interactions drive metabolic change appears to vary among individuals, indicating that host context and community composition may modulate these effects.^108-110^ Dysbiosis can distort bile acid pools and impair FXR signaling, thereby worsening steatosis and metabolic dysfunction.^32^^,^^92^ Microbiome-targeted interventions, including specific probiotics and prebiotics, have been shown in experimental models to restore bile acid composition, improve lipid handling, and reduce hepatic inflammation.^32^^,^^111^ Bile acid–microbiota crosstalk also affects cholesterol regulation, further highlighting the integrative role of the gut microbiome in liver physiology.^38^^,^^47^

A range of microbiome-centered strategies is being explored for MASLD management, including dietary modification, probiotics, prebiotics, and FMT.^32^^,^^41^ Early clinical studies report improvements in hepatic steatosis, liver enzyme profiles, and inflammatory markers following microbiome modulation.^27^^,^^94^ However, responses are heterogeneous, emphasizing the importance of personalized approaches based on individual microbial and metabolic profiles.^31^^,^^90^ Viewing MASLD through the lens of the gut–liver axis supports the microbiome as a central therapeutic target for reducing both metabolic and hepatic disease burden.^32^

### 4.4. Metabolic syndrome and hypertension

MetS is a multifactorial disorder with metabolic abnormalities that include central obesity, IR, dyslipidemia, and elevated blood pressure.^8^^,^^26^ Dysbiosis is increasingly recognized as a key contributor to MetS pathophysiology.^8^^,^^11^ Microbial communities influence host metabolism by modulating inflammatory signaling, energy balance, and production of metabolites such as SCFAs that are essential for maintaining insulin sensitivity, gut barrier integrity, and immune homeostasis.^33^

Individuals with MetS often display reduced microbial diversity and loss of health-associated taxa, which correlate with increased pro-inflammatory cytokines, IR, and other components of metabolic dysfunction.^8^^,^^9^ Depletion of *A. muciniphila* and *F. prausnitzii* is frequently observed and associates inversely with MetS severity, supporting a protective role for these species.^8^^,^^75^ Altered SCFA production profiles have been linked to impaired glycemic control and heightened inflammation,^33^^,^^35^ suggesting that quantitative and qualitative changes in microbial metabolite output are functionally important.

Microbiome-derived biomarkers are being evaluated as tools for early detection and risk stratification in MetS. Integrating microbial markers with host genetics, diet, and lifestyle factors into personalized risk models, often using machine learning approaches, may refine prediction of MetS onset and progression.^103^^,^^112^ Such models could guide individualized dietary and probiotic strategies that selectively promote beneficial microbial pathways and correct specific metabolic disturbances.^31^^,^^96^

**Figure 4.**
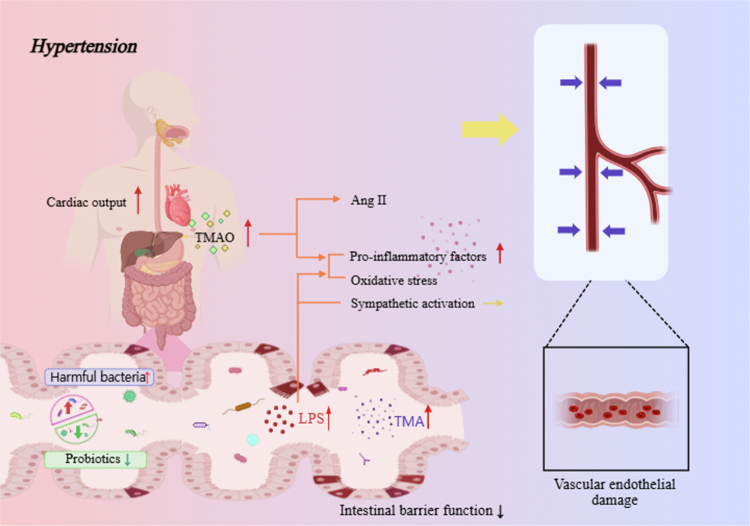
Microbiome dysbiosis, barrier disruption, and vascular dysfunction in hypertension within metabolic syndrome.

Among the clinical manifestations of MetS, hypertension is particularly significant. Growing evidence suggests that gut microbiota dysbiosis contributes to the development of hypertension through barrier disruption and maladaptive immune and neurohumoral responses.^39^ Compromised intestinal barrier integrity facilitates leakage of microbial products such as LPS and trimethylamine into the circulation.^40^^,^^63^ These molecules activate systemic inflammation, oxidative stress, and sympathetic nervous system signaling, leading to endothelial dysfunction, increased vascular resistance, and elevated blood pressure.^39^ This mechanistic framework links microbial imbalance to cardiovascular risk and highlights the potential of microbiome-directed therapies to complement conventional antihypertensive strategies (Figure 4).^31^^,^^39^

Gut microbiome dysbiosis impairs intestinal barrier integrity, allowing microbial products such as LPS and trimethylamine to translocate into the circulation. These signals drive systemic inflammation, oxidative stress, and activation of the sympathetic nervous system, which together promote endothelial dysfunction, increased vascular resistance, and elevated cardiac output. The resulting rise in blood pressure contributes to cardiovascular risk in MetS. Restoration of microbial balance, for example through diet, probiotics, or other microbiome-targeted interventions, can help preserve barrier integrity and vascular function, supporting comprehensive management of hypertension in this context.

In summary, diverse metabolic diseases, including obesity, T2D, MASLD, and MetS, share common microbiome-associated features characterized by impaired microbial diversity, reduced beneficial metabolic functions, and increased inflammatory signaling. Despite disease-specific microbial signatures, these disorders converge on a set of shared pathogenic processes involving altered metabolite production, compromised barrier integrity, immune dysregulation, and disrupted neuroendocrine communication. Advances in microbial biomarker discovery and multi-omics profiling offer increasing potential for early detection and individualized risk assessment. These insights lay the foundation for microbiome-informed, precision intervention strategies that may transform the prevention and management of metabolic diseases.

## 5. Microbiome-based intervention strategies

With the gut microbiome increasingly recognized as a key regulator of metabolic health, there is growing interest in intervention strategies that directly target microbial communities to prevent or treat metabolic disorders. A substantial body of preclinical and clinical evidence now supports the concept that deliberately reshaping the gut microbiota can favorably modify glucose homeostasis, lipid metabolism, body weight, and systemic inflammation.^6^^,^^10^^,^^95^ These insights have given rise to a spectrum of microbiome-directed approaches that range from relatively broad strategies, such as dietary modulation, to highly targeted modalities including strain-specific probiotics, synbiotics, and FMT.^41^^,^^85^^,^^113^

Dietary interventions remain foundational, since diet is a primary determinant of microbiome composition and function.^6^^,^^18^ Prebiotics and fiber-rich foods provide substrates for beneficial bacteria and promote the production of SCFAs, which support barrier integrity, modulate immunity, and improve insulin sensitivity.^33^^,^^70^ Probiotic formulations and synbiotics build on this principle by introducing live microbes, often in combination with complementary substrates, that are designed to restore or enhance specific microbial functions.^43^^,^^114^ FMT offers a more global reset of the gut ecosystem by transferring an entire microbial community from a healthy donor to a recipient with dysbiosis, with early evidence of benefit in obesity, T2D, and fatty liver disease.^27^^,^^28^^,^^41^

Beyond nutritional strategies, pharmacological and metabolite-centered approaches are emerging that leverage microbial products such as SCFAs and bile acids as signaling molecules to influence host metabolic, immune, and endocrine pathways.^33^^,^^92^ In parallel, there is increasing recognition that interindividual variability in microbiome composition and function necessitates more personalized strategies.^90^^,^^115^ Multi-omics technologies and computational tools are beginning to enable precision interventions tailored to an individual’s microbial and metabolic profile.^103^^,^^116^ Together, these developments position microbiome-based therapies as a promising complement to lifestyle and conventional pharmacological interventions in metabolic disease management.^31^^,^^85^

### 5.1. Probiotics and synbiotics

Probiotics and synbiotics have emerged as key modulators of metabolic health through their ability to restore microbial balance and influence multiple host physiological processes.^113^^,^^117^ Clinical studies have demonstrated that specific probiotic strains can improve insulin sensitivity, lipid profiles, and systemic inflammatory markers, providing a rationale for their use in conditions such as obesity, MetS, and T2D.^43^^,^^113^ Strains belonging to *Lactobacillus* and *Bifidobacterium* are among the most extensively studied and have been shown to enhance gut barrier integrity, reduce endotoxin translocation, and modulate immune responses, all of which are critical for maintaining metabolic homeostasis.^43^^,^^118^ More recently, strains such as *Limosilactobacillus reuteri (L. reuteri)* and *A. muciniphila* have attracted attention for their capacity to improve glycemic control and dyslipidemia, highlighting the potential of next-generation probiotics with more targeted metabolic effects.^11^^,^^42^

The mechanisms by which probiotics exert metabolic benefits are multifactorial. Many strains increase production of SCFAs that support epithelial health, regulate appetite and energy expenditure, and dampen inflammatory signaling.^33^ Others influence bile acid deconjugation and transformation, thereby modulating FXR and TGR5 signaling pathways that regulate glucose and lipid metabolism.^12^^,^^92^ Probiotics can also reinforce the mucus layer, upregulate tight junction proteins, and reduce gut permeability, which limits systemic exposure to pro-inflammatory microbial products such as LPS.^40^^,^^61^

Synbiotics combine probiotics with prebiotic substrates that selectively support the growth and activity of the administered strains. This design aims to generate synergistic effects, using diverse metabolic capabilities to more comprehensively address dysbiosis-associated disturbances.^113^^,^^114^ Precision synbiotic formulations that pair specific fibers or resistant starches with defined bacterial consortia have been shown to expand microbial diversity, alleviate gastrointestinal symptoms, and improve cardiometabolic parameters more effectively than single-strain products in some studies.^94^^,^^114^ Such combinations are particularly attractive for targeting complex metabolic phenotypes that involve multiple disrupted pathways.^11^^,^^113^

Importantly, clinical responses to probiotics and synbiotics are not uniform across individuals. Emerging evidence indicates that baseline gut microbiome composition, host metabolic and immune status, dietary patterns, and medication exposure may strongly influence therapeutic efficacy, giving rise to distinct responder versus non-responder phenotypes. Furthermore, many reported benefits are strain-specific, and outcomes vary across clinical trials due to differences in formulation, dosage, intervention duration, and study design, underscoring ongoing challenges in standardization and comparability.

Sustained adherence is essential for achieving durable metabolic benefits from probiotics and synbiotics. Factors that influence adherence include perceived effectiveness, ease of integration into daily routines, and access to clear educational and behavioral support.^119^^,^^120^ Individuals who experience tangible improvements in glycemic control, energy levels, or gastrointestinal comfort are more likely to maintain long-term use. Overall safety profiles are favorable, with most strains being well tolerated and only mild gastrointestinal symptoms reported in a minority of users.^113^ Nevertheless, careful strain selection and monitoring are advisable in immunocompromised or critically ill individuals.

Looking ahead, the incorporation of probiotics and synbiotics into metabolic care pathways represents a practical and scalable strategy.^31^ Future research priorities include refining strain selection based on mechanistic insight,^11^ optimizing multi-strain formulations, and integrating strain-level genomic and metabolomic data to design interventions that align more precisely with individual microbiome configurations.^103^^,^^121^ Addressing real-world adherence challenges through patient education and clinician engagement will be essential for translating their potential into consistent clinical benefit.

### 5.2. Fecal microbiota transplantation technology

FMT has gained prominence as a powerful tool for restoring gut microbial homeostasis by transferring an entire microbial community from a healthy donor to a recipient with dysbiosis. While FMT is now well established for recurrent *Clostridioides difficile* (*C. difficile*) infection, there is growing interest in its application to metabolic diseases that share common microbiome signatures, including metabolic dysfunction–associated steatotic liver disease, obesity, and T2D.^32^^,^^41^ Clinical studies have reported improvements in insulin sensitivity, hepatic steatosis, and other metabolic parameters following FMT from metabolically healthy donors, particularly in individuals with severe obesity and MetS.^27^^,^^28^

The success of FMT depends heavily on donor selection.^28^ Donors with high microbial diversity and a predominance of beneficial taxa are more likely to yield favorable metabolic effects in recipients. Accordingly, rigorous donor screening protocols that assess clinical history, infectious risk, dietary habits, and microbiome composition are essential to ensure both safety and efficacy.^41^^,^^122^ The concept of a metabolically beneficial or “super” donor has emerged from studies in which only a subset of donors confer robust metabolic benefits,^28^ underscoring the need for standardized criteria that capture functional as well as taxonomic attributes of donor microbiota.

Equally important is the monitoring of microbiota engraftment after transplantation. Longitudinal assessment of microbial colonization dynamics using high-throughput sequencing, metabolomics, and computational analysis enables evaluation of how effectively donor microbiota establish and persist in the recipient gut.^28^^,^^37^ Sustained engraftment of donor-derived taxa, particularly SCFA producers and barrier-supporting species such as *A. muciniphila*, has been linked to improved insulin sensitivity, reduced inflammation, and more favorable lipid profiles.^11^^,^^28^ Donor–recipient compatibility appears to strongly influence long-term microbiota stability and metabolic response,^28^^,^^122^ suggesting that matching based on microbial or metabolomic signatures may enhance therapeutic outcomes.^103^

Despite its promise, FMT faces several challenges in the metabolic disease context. These include defining optimal indications and timing, standardizing preparation and delivery routes, and clarifying long-term safety and durability of benefit.^41^^,^^122^ There is also a need to mitigate risks related to transfer of undesirable traits, including potential pathogens or microbiome configurations that might predispose to other diseases.^31^^,^^122^ As mechanistic understanding deepens, FMT may gradually evolve toward more refined approaches based on defined microbial consortia or synthetic microbiota that capture key beneficial functions while improving safety, reproducibility, and regulatory tractability.^31^^,^^123^^,^^124^ For now, FMT represents a compelling proof of concept that directly reprogramming the gut microbiota can modify metabolic trajectories, provided that indication selection, donor screening, and post-transplant monitoring are carefully optimized.^28^^,^^122^

### 5.3. Dietary intervention strategies

Dietary intervention is a cornerstone of microbiome modulation and a central component of strategies to improve metabolic health.^18^ Among dietary factors, fermentable fibers have a particularly prominent role. Fermentation of fibers by gut microbiota enhances microbial diversity, increases the abundance of beneficial taxa, and drives production of SCFAs such as butyrate.^33^^,^^70^ These metabolites exert anti-inflammatory effects, support gut barrier integrity, and contribute to better glycemic control and lipid handling.^33^ Higher fiber intake is consistently associated with more favorable microbiome profiles and reduced risk of obesity, T2D, and cardiovascular disease.^70^^,^^125^

The type and source of dietary fiber substantially influence microbial composition and metabolic output.^70^ Soluble fibers such as inulin and beta-glucans are more readily fermented than many insoluble fibers and often produce more pronounced metabolic benefits. Resistant starches also serve as important substrates for butyrate-producing bacteria and can improve insulin sensitivity and adiposity measures.^94^^,^^126^ These observations support the concept of personalized fiber-based interventions in which specific fiber types or combinations are matched to an individual’s baseline microbiome to maximize SCFA production, modulate immune tone, and optimize metabolic outcomes.^96^^,^^125^

Polyphenols, which are abundant in fruits, vegetables, whole grains, tea, and other plant-derived foods, are gaining recognition for their prebiotic-like effects.^127^^,^^128^ These compounds can selectively promote the growth of health-associated taxa such as *Lactobacillus* and *Bifidobacterium* while suppressing potentially pathogenic organisms.^127^^,^^129^ At the same time, polyphenols are metabolized by gut microbes into bioactive derivatives with antioxidant, anti-inflammatory, and insulin-sensitizing properties.^128^^,^^129^ This bidirectional interaction illustrates how polyphenol-rich diets can reshape microbiome composition and function, and how the microbiome in turn influences the bioactivity of dietary components.^127^^,^^130^ Integrating fiber and polyphenol strategies within overall dietary patterns that resemble non-industrialized or minimally processed diets may help recapitulate microbiome configurations associated with lower cardiometabolic risk.^18^^,^^95^

The emerging field of personalized nutrition seeks to harness microbiome data to predict and optimize individual responses to dietary interventions.^90^^,^^96^ Considerable interindividual variability exists in how people metabolize specific fibers, polyphenols, and other nutrients, and this variability is partly driven by differences in gut microbiota composition and function.^7^^,^^125^ Predictive models that incorporate baseline microbial features have been used to forecast glycemic responses and SCFA production in response to specific foods or dietary patterns, enabling the design of precision dietary recommendations.^104^^,^^131^ As these approaches are refined, they may allow clinicians to select diets that not only align with general health guidelines but also are tailored to a person’s unique microbiome in order to maximize metabolic benefit.^85^^,^^96^

### 5.4. Toward precision microbiome-based therapeutics

Personalization of microbiome-targeted interventions represents a crucial frontier in metabolic health.^85^ Interindividual variability in microbial composition, metabolic capacity, and host responses means that a given intervention can have markedly different effects across patients.^90^^,^^115^ Precision approaches aim to tailor probiotics, synbiotics, dietary modifications, pharmacological agents, or FMT protocols to an individual’s microbial and metabolic profile, thereby increasing efficacy and reducing the likelihood of adverse or neutral responses.^96^^,^^122^ To provide a comparative overview of microbiome-based therapeutic strategies and to highlight key factors contributing to inter-individual variability in treatment response, major intervention modalities are summarized in Table 1.

**Table 1.** Overview of microbiome-based therapeutic strategies for metabolic health.

Intervention type	Primary mechanisms of action	Targeted metabolic outcomes	Representative strains or approaches	Precision considerations and limitations
Probiotics	SCFA production; reinforcement of gut barrier integrity; immunomodulation; modulation of bile acid signaling	Improved insulin sensitivity; reduced inflammation; lipid profile improvement	*Lactobacillus spp.; Bifidobacterium spp.; L. reuteri;* *A. muciniphila*	Strain-specific efficacy; responder versus non-responder phenotypes; variability in formulations and dosing regimens
Synbiotics	Synergistic interactions between probiotics and tailored prebiotic substrates	Enhanced microbial diversity; improved cardiometabolic risk markers	Fiber–probiotic combinations; resistant starch-based formulations	Dependence on baseline microbiome; dietary adherence requirements; limited standardization across trials
Dietary modulation	Substrate-driven reshaping of microbial ecology; increased SCFA production	Weight reduction; improved glycemic control and lipid metabolism	High-fiber diets; polyphenol-rich dietary patterns	High inter-individual variability; long-term adherence challenges
Fecal microbiota transplantation (FMT)	Community-level ecosystem restructuring; restoration of beneficial microbial functions	Transient improvement in insulin sensitivity and metabolic inflammation	Lean-donor FMT; metabolically optimized donors	Donor selection; safety and regulatory constraints; durability of effects; risk of adverse microbial transfer

Advanced multi-omics technologies, including metagenomics, metatranscriptomics, metabolomics, and microbiomics, provide comprehensive views of microbiome–host interactions.^22^^,^^116^ By integrating these data with clinical phenotypes and environmental factors, it is possible to identify microbial taxa, metabolic pathways, and bioactive compounds associated with favorable metabolic states.^7^^,^^103^ Such information can inform the rational design of new interventions, guide the selection of existing therapies for specific patient subgroups, and help define biomarkers that predict treatment response.^104^^,^^132^

Machine learning and systems biology approaches are beginning to transform these complex datasets into actionable clinical tools.^116^^,^^132^ Predictive models that combine microbiome, dietary, and lifestyle data can help identify individuals who are likely to benefit from particular interventions,^115^^,^^131^ such as high-fiber diets, specific probiotic strains, or FMT from donors with defined microbial signatures.^28^^,^^95^

**Figure 5.**
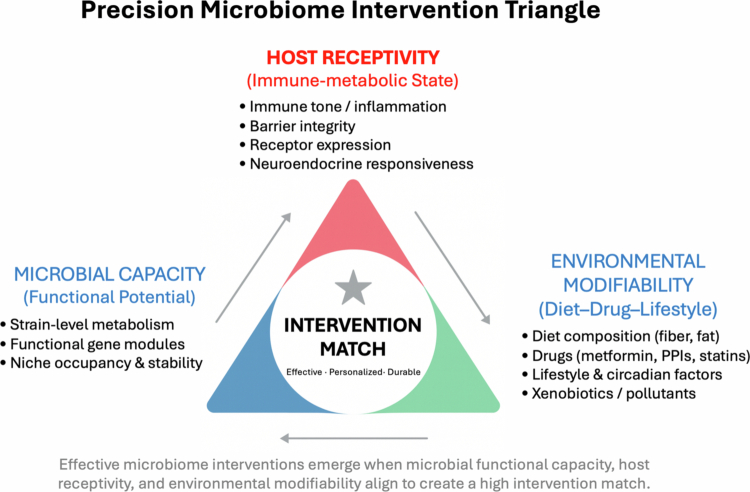
The precision microbiome intervention triangle.

Despite these advances, therapeutic responses across dietary, probiotic, synbiotic, postbiotic, and FMT strategies remain markedly heterogeneous, and clinical translation is constrained by strain-specific effects, variability across trials, and limited standardization of intervention protocols. To provide a mechanistic explanation for this variability and to guide the rational design of next-generation precision interventions, we propose a conceptual framework that integrates three fundamental determinants of microbiome therapeutic responsiveness: microbial capacity, host receptivity, and environmental modifiability. These domains collectively shape whether a given intervention can effectively modulate microbial function, engage host pathways, and ultimately produce clinically meaningful benefit. This Precision Microbiome Intervention Triangle is depicted in Figure 5 and serves as a unifying model that bridges mechanistic insight with therapeutic translation.

The Precision Microbiome Intervention Triangle provides an integrative framework to explain interindividual variability in microbiome-based therapeutic responses. Microbial capacity represents the strain-level metabolic potential, ecological stability, and functional gene networks that govern microbial output. Host receptivity encompasses the immune–metabolic state, barrier integrity, receptor expression patterns, and neuroendocrine responsiveness that determine whether microbial signals can be sensed and translated into physiological effect. Environmental modifiability reflects diet composition, medication exposures, lifestyle factors, and xenobiotics that define the ecological context in which microbes operate. Effective, personalized, and durable microbiome interventions emerge only when these three domains align to generate a high intervention match. This framework provides a mechanistic rationale for heterogeneous responses to dietary modulation, probiotics, synbiotics, postbiotics, and FMT, and outlines a conceptual foundation for precision microbiome therapeutics.

In summary, microbiome-based interventions hold substantial promise for improving metabolic health through a combination of dietary modification, biological and pharmacological modulation, and personalized treatment strategies. As the field moves from broad empiric approaches toward mechanism-based, precision therapeutics, microbiome-targeted strategies are poised to become integral components of metabolic disease management and to complement emerging frameworks for precision medicine in endocrinology and hepatology.^18^^,^^22^^,^^85^

## 6. Future research directions and challenges

Research on the gut microbiome and metabolic health is advancing rapidly, but substantial gaps remain between mechanistic insight and routine clinical application.^17^ Interindividual variability, incomplete understanding of causality, technological and analytical complexity, ethical and regulatory issues, and limited long-term clinical evidence all constrain the pace of translation.^90^^,^^133^ Addressing these challenges will be essential to realize the full potential of microbiome-based strategies for metabolic disease prevention and treatment.^16^^,^^85^

One major challenge is the pronounced interindividual variability of the microbiome, shaped by genetics, diet, medications, geography, environment, and lifestyle.^30^^,^^90^ Microbial taxa or pathways that confer metabolic benefits in one host may be neutral or even detrimental in another, depending on the broader community context and host metabolic status.^7^^,^^134^ This diversity complicates the development of standardized, “one-size-fits-all” interventions and underscores the need to pivot toward personalized strategies that explicitly incorporate individual microbiome profiles into therapeutic design.^85^^,^^96^

A second critical issue is causality. Many associations between dysbiosis and metabolic diseases such as obesity, T2D, and MASLD are based on cross-sectional human studies, which cannot determine whether microbial changes precede or follow disease onset.^14^^,^^15^ While germ-free models and FMT provide compelling causal evidence in animals,^6^^,^^42^ the directionality and strength of these relationships in humans remain less clear.^21^^,^^28^ Large, longitudinal cohort studies and well-controlled interventional trials will be needed to map temporal dynamics of microbiome change across the natural history of metabolic disorders and to define windows during which microbiome modulation is most effective.^104^^,^^135^

Translating complex microbiome science into practical clinical tools represents another major hurdle. Differences in sample collection, storage, DNA extraction, sequencing platforms, and bioinformatic pipelines all affect microbiome readouts, limiting comparability across studies and complicating meta-analysis.^135^ In this context, future studies could align around a minimum core dataset that routinely captures key dietary, medication, and metabolic variables (e.g., diet, medication use, BMI, glycemic status, antibiotic history, sampling timing, and geographic context). Harmonized sample collection and processing, including standardized sampling materials, cold-chain handling, DNA extraction, and sequencing platforms, are equally critical. Finally, adoption of reproducible, open-source analytic pipelines and shared reference databases would markedly improve cross-study comparability. Standardized protocols and quality control frameworks are urgently needed to ensure reproducibility and to support regulatory approval of microbiome-based diagnostics and therapeutics.^31^^,^^133^ At present, interventions such as probiotics, prebiotics, dietary modification, and FMT show variable efficacy across clinical trials, highlighting the need for rigorous study design, appropriate control groups, and clear definition of responder subgroups.^28^^,^^136^

Ethical and regulatory considerations add further complexity.^133^ Microbiome data raise important questions regarding informed consent, data privacy, ownership, and secondary use of genomic and metagenomic information.^133^^,^^137^^,^^138^ Microbiome-targeted therapies, particularly FMT and engineered microbial products, may carry unforeseen long-term risks, including off-target ecological effects or unintended immune responses.^41^^,^^139^ Existing regulatory frameworks were not designed for dynamic, personalized, living therapeutics and must evolve to accommodate adaptive trial designs, post-marketing surveillance, and safety monitoring tailored to microbiome-based interventions.^133^^,^^139^ Robust ethical guidelines and engagement of diverse stakeholders, including patients, clinicians, regulators, and ethicists, will be crucial to ensure responsible innovation.^133^

Overall, future progress will depend on integrative, multidisciplinary efforts that combine longitudinal human studies, mechanistic experimentation, advanced analytics, and careful clinical translation.^22^^,^^116^ The following sections highlight specific research directions and structural challenges that must be addressed to advance microbiome-informed metabolic medicine.

### 6.1. Species- and strain-specific microbial research

Understanding microbiome–host interactions at species and strain level is a central priority for future research.^87^^,^^134^ Community-level metrics such as diversity or broad taxonomic shifts provide important context but often lack the resolution needed to explain functional outcomes.^21^^,^^134^ Increasing evidence shows that different strains within the same species can exhibit distinct metabolic capacities and immunomodulatory properties, leading to divergent effects on host physiology.^121^^,^^140^

Recent work has begun to dissect the molecular mechanisms through which specific microbial strains modulate host metabolism.^87^^,^^141^ Some gut bacteria influence host drug metabolism by altering cytochrome P450 enzyme activity, thereby changing the pharmacokinetics and efficacy of commonly used medications.^142^^,^^143^ Other studies highlight the role of the microbiome in circadian regulation of metabolism,^73^ showing that the timing of microbial activity and metabolite production can affect energy balance, insulin sensitivity, and susceptibility to metabolic disorders.^33^^,^^73^ These findings point to the importance of temporal dynamics, in addition to taxonomic composition, when assessing microbiome contributions to metabolic health.

Strain-level differences are particularly evident within genera such as *Bifidobacterium* and *Lactobacillus.*^121^^,^^140^ Distinct strains can differ in SCFA production, indole and other tryptophan derivatives, bile acid transformation, and other bioactive metabolites that shape the gut–liver axis, immune responses, and inflammatory tone.^12^^,^^121^ In MASLD models, for example, specific *Bifidobacterium* strains have been shown to differentially modulate hepatic fat accumulation and inflammatory markers.^29^^,^^144^ Such observations reinforce that not all members of a species are functionally equivalent and that future probiotic and synbiotic design must account for strain-level specificity.^96^^,^^121^

Cross-species comparisons between animal models and humans offer another important direction.^6^^,^^37^ Aligning microbial signatures and metabolic phenotypes across species can identify conserved pathways that are more likely to represent robust therapeutic targets.^87^^,^^134^ Systems-level tools and network-based approaches now allow visualization of microbiome metabolic circuits and their links to host pathways, facilitating the discovery of high-value nodes for intervention.^145^^,^^146^ Continued investment in species- and strain-resolved multi-omics, combined with mechanistic validation, will be essential for moving from descriptive associations to precise, mechanistically grounded therapeutics.^22^^,^^103^

### 6.2. Barriers to clinical translation

Despite impressive mechanistic advances, the translation of microbiome research into metabolic medicine remains challenging. One major barrier is the persistent ambiguity surrounding causality in human studies.^17^^,^^22^ Many reported associations between dysbiosis and metabolic traits rely on cross-sectional analyzes, leaving open whether microbial changes are drivers, modifiers, or consequences of disease.^85^ Longitudinal cohorts, interventional trials, and Mendelian randomization approaches will be critical to disentangle these relationships and to define which microbial features represent viable therapeutic targets versus secondary markers of disease.^30^

Standardization is another core issue. Heterogeneity in study design, patient selection, diet and medication control, and microbiome analytic methods all contribute to inconsistent findings.^116^^,^^133^ Without harmonized protocols for sample collection, processing, sequencing, and bioinformatics, it is difficult to compare results across trials or to pool data for meta-analysis.^147^ International consortia, shared reference standards, and consensus guidelines for microbiome research are needed to enhance reproducibility and regulatory confidence.

The evidence base for microbiome-targeted therapies in metabolic disease, while promising, remains incomplete. Probiotics, prebiotics, synbiotics, dietary interventions, and FMT have each shown benefits in selected trials, but effect sizes are often modest and responses highly variable.^28^^,^^41^ Many studies are small, short-term, or lack stratification by baseline microbiome, genetics, or lifestyle factors that could influence efficacy.^85^^,^^115^ Robust, adequately powered, randomized controlled trials with long-term follow-up are necessary to define which interventions work, in whom, and for how long. This includes careful assessment of durability of benefit, potential need for maintenance regimens, and the possibility of rebound dysbiosis or compensatory ecological changes.

Safety and regulation also represent significant translational barriers. Short-term use of probiotics and dietary interventions appears generally safe in most populations, but the long-term consequences of sustained microbiome modulation are poorly understood.^85^^,^^139^ NNS, for example, can alter microbial composition, yet their chronic metabolic and microbiome impacts remain uncertain.^57^^,^^58^ FMT and engineered microbial therapies carry additional risks related to pathogen transmission, off-target colonization, and unforeseen ecological effects.^122^^,^^133^ Regulatory frameworks must evolve to accommodate the unique properties of living therapeutics, including adaptive trial designs, rigorous donor and product screening, pharmacovigilance, and post-marketing surveillance.^133^

Ethical and social considerations further complicate translation. Microbiome data are deeply personal and may reveal information about disease risk, ancestry, or lifestyle.^90^^,^^148^ Ensuring informed consent, data privacy, responsible data sharing, and equitable access to emerging microbiome-based therapies will be essential.^90^ Designing inclusive studies that reflect diverse populations is critical, both to ensure generalizability and to avoid exacerbating existing health disparities as precision microbiome medicine matures.^89^^,^^135^

### 6.3. Technological innovation needs

Realizing the potential of microbiome-based precision medicine will require continued technological innovation across multiple domains.^22^ At the analytic level, integration of multi-omics data, including metagenomics, metatranscriptomics, metaproteomics, metabolomics, and host genomics, remains a complex challenge.^103^^,^^116^ Harmonizing these datasets demands advanced computational frameworks, standardized pipelines, and transparent, interpretable machine learning models that can move beyond correlation to actionable prediction.^149^

Gene-editing and microbiome engineering tools represent another frontier. CRISPR-based systems and related technologies now allow targeted modification of microbial genomes, raising the possibility of “editing” resident strains to enhance beneficial functions such as SCFA production or to suppress pathways that promote inflammation or metabolic dysfunction.^123^^,^^124^ Proof-of-concept studies demonstrate that microbial gene editing can influence host metabolic outcomes in experimental models, but clinical translation will require rigorous safety evaluation, ethical oversight, and regulatory adaptation to address the unique risks of modifying live microbial communities in humans.^123^^,^^133^

Dynamic monitoring technologies are also needed to capture the temporal dimension of host–microbiome interactions.^116^^,^^149^ Current approaches largely rely on intermittent stool sampling, which provides limited insight into real-time microbial and metabolic dynamics. Next-generation tools could include minimally invasive sampling methods, time-series multi-omics, and digital health platforms that combine dietary and lifestyle tracking with microbiome readouts. Wearable sensors and mobile applications, linked to predictive models, may ultimately enable personalized feedback loops in which individuals receive real-time guidance on diet or therapy based on their evolving microbiome and metabolic status.^150^

Finally, large-scale data infrastructure and collaborative platforms will be essential to manage and interpret the expanding volume of microbiome and metabolic data. Big-data analytics, systems biology, and artificial intelligence can help identify robust microbial signatures of metabolic disease, predict individual responses to interventions, and optimize trial design.^103^^,^^116^^,^^132^ Shared databases, interoperable formats, and open-science initiatives will accelerate discovery and ensure that insights can be replicated and extended across cohorts and populations.

### 6.4. Outlook

Advancing microbiome-mediated metabolic health will require coordinated progress on multiple fronts: deeper species- and strain-resolved mechanistic understanding, longitudinal human studies to establish causality, standardized and ethically sound clinical research frameworks, and technological innovations that support real-time monitoring and complex data integration.^22^ By addressing these challenges, the field can move from descriptive cataloging of dysbiosis toward precise, mechanistically informed interventions that are tailored to the unique microbial and metabolic profile of each individual. In this vision, microbiome-based strategies will not only complement existing lifestyle and pharmacological treatments but may ultimately help redefine how metabolic diseases are prevented, diagnosed, and managed.^72^

## 7. Conclusions and clinical implications

The gut microbiome has emerged as a fundamental regulator of metabolic health, influencing host physiology through an integrated network of microbial metabolites, barrier and immune modulation, and neuroendocrine signaling along the microbiota–gut–brain–pancreas axis. Across obesity, T2D, MASLD, MetS, and hypertension, diverse lines of evidence converge on a shared mechanistic framework in which dysbiosis disrupts metabolic homeostasis, promotes chronic low-grade inflammation, and accelerates cardiometabolic risk. At the same time, interindividual heterogeneity in microbiome composition and function helps explain why metabolic disease phenotypes and responses to lifestyle or pharmacologic therapies vary so widely between patients.

These insights carry important clinical implications. First, they position the gut microbiome as both a biomarker reservoir and a modifiable therapeutic target in metabolic disease. Microbial taxa, metabolites, and functional signatures hold promise for improving early detection, refining risk stratification, and enabling more accurate prediction of disease trajectories. Second, they provide a rationale for incorporating microbiome-directed strategies, such as targeted probiotics and synbiotics, dietary modulation, and, in selected cases, FMT, into comprehensive management plans for metabolic disorders. While these interventions are unlikely to replace established therapies, they may meaningfully augment lifestyle and pharmacological approaches by addressing upstream ecological drivers of metabolic dysfunction.

At the same time, translation into routine care must proceed cautiously. The evidence base for microbiome-based therapies in metabolic disease remains heterogeneous, with substantial variability in study design, sample size, duration, and outcome measures. Many reported benefits are modest and context-dependent, and long-term safety data are limited, particularly for more intensive interventions such as FMT or engineered microbial products. Robust randomized controlled trials, standardized protocols, and careful patient selection will be critical to determine which microbiome-targeted strategies offer clinically meaningful benefit, for which patients, and under what conditions. Ethical and regulatory frameworks must also evolve to address data privacy, equitable access, and the unique properties of living therapeutics.

Looking forward, integration of microbiome profiling into precision metabolic medicine offers a compelling path toward more individualized care. As multi-omics platforms and computational tools become more accessible, clinicians may increasingly be able to incorporate microbial information alongside genetic, metabolic, and lifestyle data to guide preventive and therapeutic decisions. In this vision, microbiome-informed nutrition plans, strain-specific probiotics, and tailored microbiome-modulating regimens could be selected based on an individual’s baseline microbial configuration and monitored over time to optimize metabolic outcomes.

In conclusion, the gut microbiome is now recognized as a central node in the network that links environment, lifestyle, and host biology to metabolic health and disease. Continued progress will depend on closing key gaps in causal understanding, refining and validating microbiome-based interventions, and building scalable frameworks for their safe and equitable deployment. If these challenges can be met, microbiome-focused strategies have the potential not only to enhance current approaches to obesity, diabetes, fatty liver disease, and MetS, but also to reshape how metabolic diseases are conceptualized, prevented, and treated in the era of precision medicine.

## Data Availability

No datasets were generated or analyzed during the current study.

## Abbreviations

CNS: Central nervous system
FMT: Fecal microbiota transplantation
FXR: Farnesoid X receptor
GABA: Gamma-aminobutyric acid
GPCRs: G-protein-coupled receptors
IR: Insulin resistance
LPS: Lipopolysaccharides
MASH: Metabolic dysfunction-associated steatohepatitis
MASLD: Metabolic dysfunction-associated steatotic liver disease
MetS: Metabolic syndrome
NAFLD: Non-alcoholic fatty liver disease
NASH: Non-alcoholic steatohepatitis
NNS: Non-nutritive sweeteners
OTUs: Operational taxonomic units
SCFAs: Short-chain fatty acids
SCI: Spinal cord injury
T2D: Type 2 diabetes
